# Racial and Ethnic Disparities in Acute Myocarditis Outcomes in the United States

**DOI:** 10.1002/clc.70460

**Published:** 2026-08-31

**Authors:** Maria F. Osorio, Paola Lecompte‐Osorio, Sebastian Vasquez‐Ariza, Michael Dangl, Louis Vincent, Johanna Contreras, Rosario Colombo

**Affiliations:** ^1^ University of Miami/Jackson Memorial Hospital Miami Florida USA; ^2^ Cardiovascular Center Boston University School of Medicine, Boston Medical Center Boston Massachusetts USA; ^3^ Department of Medicine Universidad del Norte Barranquilla Colombia; ^4^ Division of Cardiovascular Medicine University of Florida, College of Medicine Gainesville Florida USA; ^5^ Department of Cardiovascular Medicine, Wake Forest University School of Medicine Sanger Heart and Vascular Institute, Atrium Health Charlotte North Carolina USA; ^6^ Mount Sinai Fuster Heart Hospital Icahn School of Medicine at Mount Sinai, Mount Sinai Hospital New York New York USA; ^7^ Division of Cardiovascular Medicine Miller School of Medicine, University of Miami Health System Miami Florida USA

**Keywords:** acute myocarditis, cardiogenic shock, ethnic disparities, in‐hospital mortality, mechanical circulatory support, racial disparities

## Abstract

**Background:**

Racial and ethnic disparities in cardiovascular outcomes are well established, but whether these inequities extend to acute myocarditis remains insufficiently characterized.

**Hypothesis:**

Racial and ethnic minority groups hospitalized with acute myocarditis would have higher odds of in‐hospital mortality than White patients.

**Methods:**

Using the National Inpatient Sample from 2012 to 2021, we identified adult hospitalizations with a primary diagnosis of acute myocarditis. The primary outcome was all‐cause in‐hospital mortality. Secondary outcomes included cardiogenic shock, mechanical circulatory support (MCS), cardiac arrest, ventricular tachycardia/fibrillation (VT/VF), acute kidney injury, renal replacement therapy, and stroke. Multivariable logistic regression estimated adjusted odds ratios (aORs).

**Results:**

A total of 105 235 weighted hospitalizations were identified. After adjustment, all minority groups had higher odds of in‐hospital mortality than White patients: Black (aOR 1.30, 95% CI 1.10–1.55), Hispanic (aOR 1.37, 95% CI 1.14–1.66), Asian/Pacific Islander (aOR 2.07, 95% CI 1.58–2.72), and Other (aOR 1.40, 95% CI 1.07–1.83). Asian/Pacific Islander patients had the highest odds of cardiogenic shock and MCS use. Black patients had higher odds of cardiac arrest and VT/VF. Hispanic patients had higher in‐hospital mortality despite lower odds of VT/VF and MCS use. Race and ethnicity by pandemic era interaction for mortality was not significant (*p* = 0.072).

**Conclusions:**

Among adults hospitalized with acute myocarditis, racial and ethnic minority groups had higher adjusted odds of in‐hospital mortality than White patients. Distinct patterns of complications further underscore the need for equitable, attentive, and risk‐informed clinical care.

## Graphical abstract

In a nationally representative analysis of 105 235 U.S. hospitalizations for acute myocarditis, racial and ethnic minority groups had higher adjusted in‐hospital mortality than White patients. Asian/Pacific Islander patients had the greatest mortality and hemodynamic risk, while Black and Hispanic patients showed distinct complication patterns.

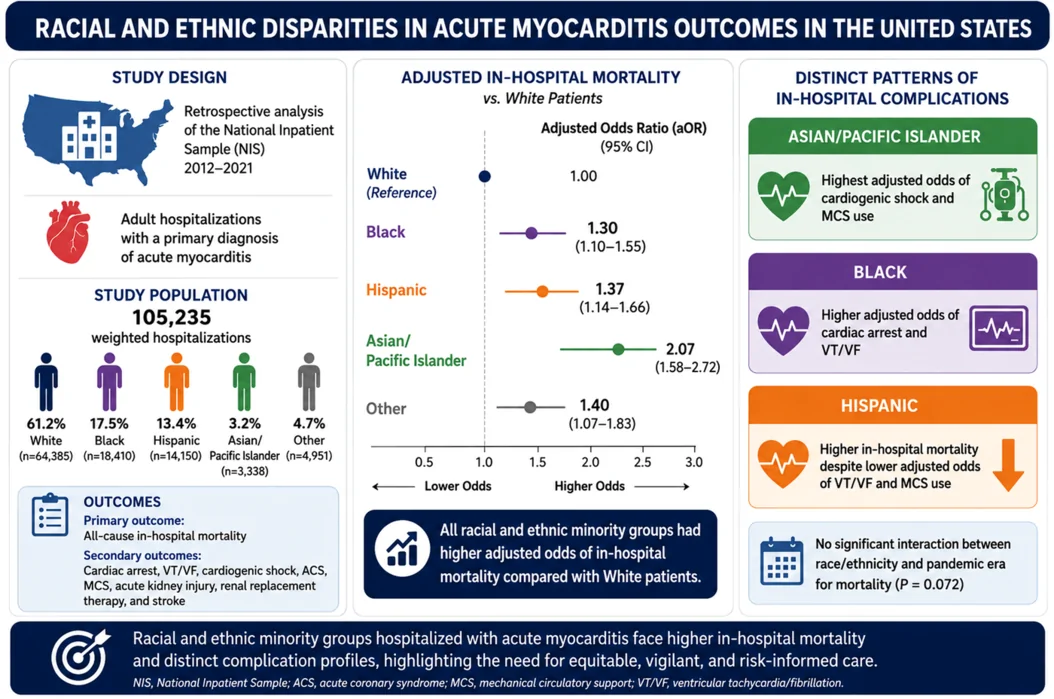

**Abbreviations**
ACS: acute coronary syndrome
AHF: acute heart failure
AHRQ: Agency for Healthcare Research and Quality
AKI: acute kidney injury
aOR: adjusted odds ratio
ARIC: Atherosclerosis Risk in Communities
CAD: coronary artery disease
CHF: chronic heart failure
CI: confidence interval
CKD: chronic kidney disease
COPD: chronic obstructive pulmonary disease
COVID‐19: coronavirus disease 2019
CS: cardiogenic shock
DVT/PE: deep vein thrombosis/pulmonary embolism
ECMO: extracorporeal membrane oxygenation
ESRD: end‐stage renal disease
HCUP: Healthcare Cost and Utilization Project
ICD: implantable cardioverter‐defibrillator
ICD‐10‐CM: International Classification of Diseases, Tenth Revision, Clinical Modification
ICD‐9‐CM: International Classification of Diseases, Ninth Revision, Clinical Modification
MCS: mechanical circulatory support
MI: myocardial infarction
NIS: National Inpatient Sample
OR: odds ratio
PAD: peripheral artery disease
RRT: renal replacement therapy
SCD: sudden cardiac death
SNF: skilled nursing facility
VT/VF: ventricular tachycardia/fibrillation

## 1 Introduction

Cardiovascular disease remains the leading cause of mortality in the United States, accounting for 919 032 deaths in 2023 [1]. Racial, ethnic, and sex‐based disparities in cardiovascular healthcare access, treatment, and outcomes have been consistently documented over several decades, reflected in the disproportionate burden of cardiovascular risk factors and adverse events among minority populations [2]. Minority populations experience disproportionate burdens of cardiovascular risk factors and adverse events, including hypertension, myocardial infarction (MI), acute heart failure (AHF), stroke, cardiogenic shock outcomes, and cardiometabolic disease across Black, Hispanic, and South Asian populations [3, 4, 5, 6, 7].

Whether these disparities extend to less common but potentially life‐threatening cardiovascular conditions, such as acute myocarditis, remains poorly characterized. Acute myocarditis spans a wide clinical spectrum from mild chest pain and subclinical ventricular dysfunction to AHF, malignant arrhythmias, cardiogenic shock, and accounts for 2%–9% of sudden cardiac deaths (SCD) among athletes in both US and European registries [8, 9]. Contemporary global burden analyses estimate an increase from 320 623 to 505 030 cases from 1990 to 2021 among 204 different countries [10].

Despite its clinical significance, data examining racial and ethnic differences in myocarditis outcomes remain limited, with prior work largely restricted to pediatric populations or coronavirus disease 2019 (COVID‐19) associated myocarditis [11, 12, 13]. To address this gap, we analyzed the National Inpatient Sample (NIS) to examine associations between race and ethnicity and in‐hospital outcomes; including mortality, cardiogenic shock, mechanical circulatory support (MCS), arrhythmias, renal complications, and stroke, among adults hospitalized with acute myocarditis in the United States from 2012 to 2021, and to identify demographic and socioeconomic factors associated with these disparities.

## 2 Methods

### 2.1 Study Design

This cross‐sectional retrospective study used the NIS from January 1, 2012, to December 31, 2021. The NIS is a nationally representative all‐payer inpatient database developed through Healthcare Cost and Utilization Project (HCUP) of the Agency for Healthcare Research and Quality (AHRQ) [14]. The database captures information on primary and secondary diagnoses, patient demographics, primary payer, comorbidities, length of stay, and in‐hospital mortality Because the database is public and de‐identified, the study was exempt from institutional review board oversight. The investigation was conducted in accordance with the ethical principles of the Declaration of Helsinki.

### 2.2 Study Population

Adult hospitalizations among patients aged 18 years or older with a principal diagnosis of acute myocarditis were identified using International Classification of Diseases, Ninth Revision, Clinical Modification (ICD‐9‐CM), and Tenth Revision, Clinical Modification (ICD‐10‐CM), codes, as listed in Supporting Information S1: Table 1. Each NIS entry represents a hospitalization rather than a unique patient.

### 2.3 Exposures, Covariates, and Outcomes

The primary exposure was race and ethnicity, categorized according to NIS definitions as White, Black, Hispanic, Asian/Pacific Islander, and Other. For purposes of this study, Native Americans were included in the “Other” category. Baseline covariates included age, sex, primary payer, median household income quartile by patient ZIP code, and comorbid conditions identified using diagnosis codes, including hypertension, obesity, diabetes mellitus, chronic obstructive pulmonary disease (COPD), coronary artery disease (CAD), chronic kidney disease (CKD), end‐stage renal disease (ESRD), peripheral artery disease (PAD), chronic heart failure (CHF), atrial fibrillation, chronic liver disease, malignancy, sepsis, anemia, and deep vein thrombosis/pulmonary embolism (DVT/PE).

The primary outcome was all‐cause in‐hospital mortality. Secondary outcomes included cardiac arrest, ventricular tachycardia/ventricular fibrillation (VT/VF), cardiogenic shock, acute coronary syndrome (ACS), receipt of mechanical circulatory support (MCS), acute kidney injury (AKI), renal replacement therapy (RRT), and stroke. MCS was defined as use of an intra‐aortic balloon pump, extracorporeal membrane oxygenation, percutaneous left ventricular assist device, biventricular assist device, or durable left ventricular assist device. Additional resource utilization outcomes included discharge disposition, length of stay, and total hospital charges.

Overall mortality rate and trends of racial and ethnic disparities in mortality rate per year (2012–2021) were described. As sensitivity analyses, we repeated the primary mortality model by differentiating hospitalizations from 2012–2019 to 2020–2021 to assess potential confounding related to coronavirus disease 2019 (COVID‐19)‐era myocarditis coding and care delivery, and we performed an interaction analysis between race and the pandemic era to determine whether disparities changed significantly over time. Also, sensitivity analyses by excluding COVID‐19 hospitalizations were performed for the mortality trends, as well as the primary adjusted model.

### 2.4 Statistical Analysis

All analyses followed AHRQ recommendations for the NIS, incorporating discharge weights, hospital strata, and clustering to generate nationally representative estimates and account for the complex survey design. Missing data were handled using multiple imputation with 10 iterations, including race, age, primary expected payer, total hospital charges, patient location, sex, and mortality; pooled estimates were combined using Rubin's rules. A complete‐case sensitivity analysis was also performed.

Continuous variables were reported as mean ± SD or median [IQR], as appropriate, and categorical variables as frequencies and percentages. Baseline characteristics and resource utilization were compared across racial groups using Pearson chi‐square tests for categorical variables and one‐way ANOVA or Kruskal‐Wallis tests for continuous variables.

Univariable and multivariable logistic regression models estimated odds ratios and adjusted odds ratios with 95% CIs for the association between race and binary in‐hospital outcomes, using White patients as the reference group. Multivariable models included prespecified clinically relevant covariates: age, sex, primary payer, median household income quartile, hypertension, obesity, diabetes mellitus, COPD, CAD, CKD, ESRD, CHF, atrial fibrillation, chronic liver disease, malignancy, anemia, history of MI, and DVT/PE. A two‐sided *p*‐value < 0.05 was considered statistically significant. Analyses were performed using IBM SPSS Statistics version 31.0, and figures were created in R version 4.3.3.

## 3 Results

### 3.1 Baseline Demographics and Clinical Characteristics

**FIGURE 1:**
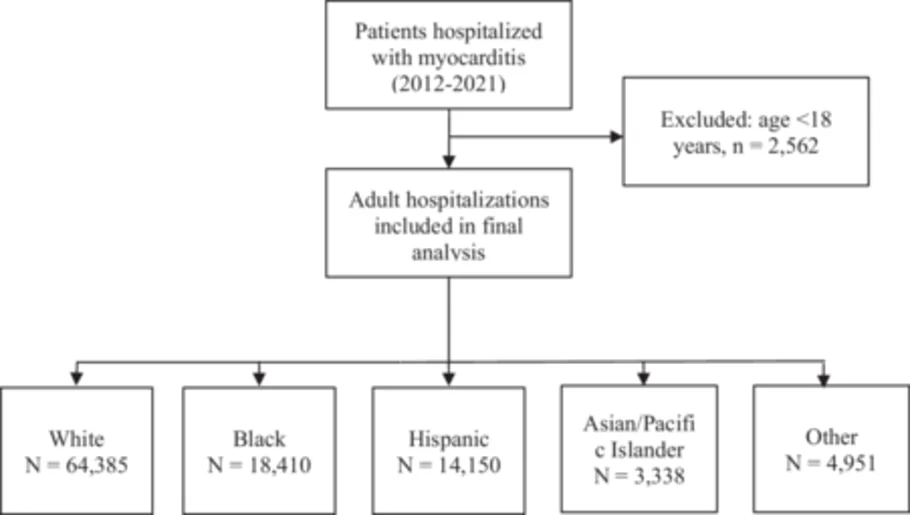
Flowchart of patient selection. Flow diagram showing selection of adult hospitalizations with a principal diagnosis of acute myocarditis from the National Inpatient Sample between 2012 and 2021. After excluding patients younger than 18 years, the final cohort included 21 047 unweighted hospitalizations, corresponding to 105 235 weighted national hospitalizations, stratified by race and ethnicity.

The study included 21 047 unweighted hospitalizations with myocarditis, corresponding to 105 235 weighted national hospitalizations. Of these, 64 385 were White, 18 410 Black, 14 150 Hispanic, 3338 Asian/Pacific Islander, and 4951 Other race/ethnicity (Figure 1). Baseline characteristics differed significantly across racial and ethnic groups (all *p* < 0.001) (Table 1). Black and Hispanic patients were younger than White patients, while female sex was more frequent among Black and Asian/Pacific Islander patients. Comorbidity patterns also differed: Black patients had the highest prevalence of hypertension, CKD, and CHF; Asian/Pacific Islander patients had the highest prevalence of diabetes and sepsis; and White patients had higher rates of COPD, CAD, atrial fibrillation, and malignancy. Insurance status and neighborhood income also varied, with Black and Hispanic patients more frequently insured through Medicaid and residing in lower‐income quartiles.

**TABLE 1:** Baseline characteristics of adults hospitalized with acute myocarditis, stratified by race and ethnicity.

Baseline Characteristics (*n* = 105 235)	White (*n* = 64 385)	Black (*n* = 18 410)	Hispanic (*n* = 14 150)	Asian/Pacific Islander (*n* = 3338)	Other (*n* = 4951)	*p*‐value
Age	50.08 (± 19.52)	44.49 (± 17.37)	42.49 (± 17.87)	49.73 (± 18.93)	44.78 (± 18.50)	< 0.001
Female	25 763 (40.0%)	8197 (44.5%)	5407 (38.2%)	1536 (46.0%)	1921 (38.8%)	< 0.001
Hypertension	27 774 (43.1%)	9900 (53.8%)	5487 (38.8%)	1592 (47.7%)	1730 (34.9%)	< 0.001
Obesity	9982 (15.5%)	3709 (20.1%)	2815 (19.9%)	429 (12.9%)	743 (15.0%)	< 0.001
Diabetes Mellitus	10 253 (15.9%)	3994 (21.7%)	2879 (20.3%)	812 (24.3%)	876 (17.7%)	< 0.001
COPD	6061 (9.4%)	1074 (5.8%)	470 (3.3%)	131 (3.9%)	312 (6.3%)	< 0.001
Coronary artery disease	14 636 (22.7%)	2880 (15.6%)	2121 (15.0%)	631 (18.9%)	896 (18.1%)	< 0.001
History of MI	3018 (4.7%)	915 (5.0%)	536 (3.8%)	116 (3.5%)	208 (4.2%)	< 0.001
Chronic kidney disease	6441 (10.0%)	3303 (17.9%)	1451 (10.3%)	515 (15.4%)	459 (9.3%)	< 0.001
End‐Stage Renal Disease	865 (1.3%)	895 (4.9%)	433 (3.1%)	148 (4.4%)	88 (1.8%)	< 0.001
Peripheral artery disease	1639 (2.5%)	356 (1.9%)	225 (1.6%)	57 (1.7%)	111 (2.2%)	< 0.001
Chronic heart failure	17 839 (27.7%)	6615 (35.9%)	3543 (25.0%)	1171 (35.1%)	1430 (28.9%)	< 0.001
Atrial fibrillation	9460 (14.7%)	1550 (8.4%)	1104 (7.8%)	425 (12.7%)	505 (10.2%)	< 0.001
Chronic liver disease	550 (0.9%)	132 (0.7%)	181 (1.3%)	30 (0.9%)	30 (0.6%)	< 0.001
Malignancy	4953 (7.7%)	914 (5.0%)	610 (4.3%)	201 (6.0%)	275 (5.6%)	< 0.001
Sepsis	8575 (13.3%)	2944 (16.0%)	2608 (18.4%)	819 (24.5%)	868 (17.5%)	< 0.001
Anemia	20 242 (31.4%)	5038 (27.4%)	2828 (20.0%)	850 (25.5%)	1051 (21.2%)	< 0.001
DVT/PE	2302 (3.6%)	880 (4.8%)	611 (4.3%)	151 (4.5%)	235 (4.7%)	< 0.001
Admission type						
Elective	3499 (5.4%)	738 (4.0%)	523 (3.7%)	133 (4.0%)	295 (6.0%)	< 0.001
Nonelective	60 763 (94.4%)	17 626 (95.7%)	13 605 (96.1%)	3199 (95.8%)	4651 (93.9%)	
Expected primary payer						
Medicare	18 532 (28.8%)	3945 (21.4%)	2134 (15.1%)	776 (23.2%)	885 (17.9%)	< 0.001
Medicaid	7563 (11.7%)	5127 (27.8%)	4248 (30.0%)	694 (20.8%)	1276 (25.8%)	
Private insurance	32 125 (49.9%)	6501 (35.3%)	5094 (36.0%)	1602 (48.0%)	2052 (41.4%)	
Self‐pay	3844 (6.0%)	1899 (10.3%)	1943 (13.7%)	144 (4.3%)	426 (8.6%)	
No charge	287 (0.4%)	176 (1.0%)	202 (1.4%)	30 (0.9%)	56 (1.1%)	
Other	2033 (3.2%)	760 (4.1%)	528 (3.7%)	90 (2.7%)	256 (5.2%)	
Median household income						
Quartile 1	12 716 (19.8%)	8235 (44.7%)	4749 (33.6%)	434 (13.0%)	1210 (24.4%)	< 0.001
Quartile 2	16 647 (25.9%)	4335 (23.5%)	3679 (26.0%)	654 (19.6%)	1193 (24.1%)	
Quartile 3	17 014 (26.4%)	3250 (17.7%)	3309 (23.4%)	893 (26.8%)	1163 (23.5%)	
Quartile 4	18 008 (28.0%)	2590 (14.1%)	2412 (17.0%)	1357 (40.7%)	1384 (28.0%)	

*Note:* Values are presented as mean ± SD or *n* (%), as appropriate. Percentages are weighted national estimates. Race and ethnicity categories reflect National Inpatient Sample reporting. The Other category includes race/ethnicity groups not otherwise specified, including Native American patients.

Abbreviations: COPD, chronic obstructive pulmonary disease; DVT/PE, deep vein thrombosis/pulmonary embolism; MI, myocardial infarction; SD, standard deviation.

### 3.2 Outcomes by Race and Ethnicity

#### 3.2.1 Unadjusted Analysis

In unadjusted analyses, Asian/Pacific Islander patients had higher odds of in‐hospital mortality compared with White patients (OR 2.04; 95% CI 1.58–2.63; *p* < 0.001). Black patients (OR 1.15; 95% CI 0.99–1.33; *p* = 0.053), Hispanic patients (OR 1.12; 95% CI 0.94–1.32; *p* = 0.176), and patients categorized as Other race/ethnicity (OR 1.20; 95% CI 0.93–1.56; *p* = 0.147) also had numerically higher unadjusted mortality than White patients, although these differences were not statistically significant (Table 2).

**TABLE 2:** Unadjusted odds ratios for in‐hospital outcomes by race and ethnicity.

Outcome	Black OR (95% CI)	*p* value	Hispanic OR (95% CI)	*p* value	Asian/Pacific Islander OR (95% CI)	*p* value	Other OR (95% CI)	*p* value
Primary Outcome								
In‐hospital mortality	1.15 (0.99–1.33)	0.053	1.12 (0.94–1.32)	0.176	2.04 (1.58–2.63)	< 0.001	1.20 (0.93–1.56)	0.147
Secondary Outcomes								
Cardiac arrest	1.38 (1.14–1.67)	< 0.001	1.02 (0.81–1.29)	0.828	1.32 (0.89–1.96)	0.164	1.15 (0.81–1.62)	0.423
Ventricular tachycardia/fibrillation	1.11 (0.98–1.26)	0.078	0.65 (0.55–0.76)	< 0.001	1.17 (0.92–1.50)	0.189	0.89 (0.71–1.13)	0.355
Cardiogenic shock	1.18 (1.04–1.34)	0.010	1.06 (0.91–1.23)	0.390	2.14 (1.71–2.68)	< 0.001	1.30 (1.04–1.62)	0.019
Acute coronary syndrome	0.91 (0.83–1.00)	0.076	0.98 (0.88–1.08)	0.719	0.98 (0.81–1.19)	0.878	1.08 (0.92–1.26)	0.323
Receipt of MCS	1.22 (1.01–1.46)	0.032	0.75 (0.58–0.96)	0.023	2.22 (1.64–3.02)	< 0.001	1.28 (0.93–1.77)	0.127
Acute kidney injury	1.71 (1.57–1.86)	< 0.001	1.06 (0.96–1.17)	0.225	1.66 (1.40–1.97)	< 0.001	1.09 (0.92–1.28)	0.288
Renal replacement therapy	2.22 (1.87–2.63)	< 0.001	1.79 (1.46–2.19)	< 0.001	2.57 (1.85–3.56)	< 0.001	1.10 (0.77–1.58)	0.583
Stroke	1.18 (0.93–1.49)	0.155	0.77 (0.56–1.05)	0.100	1.71 (1.13–2.58)	< 0.001	1.27 (0.85–1.91)	0.234

*Note:* Odds ratios are shown relative to White patients as the reference group. Values are presented as unadjusted ORs with 95% CIs. Asian/Pacific Islander was analyzed as a single category according to National Inpatient Sample reporting. The Other category includes race/ethnicity groups not otherwise specified, including Native American patients.

Abbreviations: AKI, acute kidney injury; CI, confidence interval; MCS, mechanical circulatory support; OR, odds ratio; RRT, renal replacement therapy; VT/VF, ventricular tachycardia/fibrillation.

Among secondary outcomes, Black patients had higher unadjusted odds of cardiac arrest (OR 1.38; 95% CI 1.14–1.67; *p* < 0.001), cardiogenic shock (OR 1.18; 95% CI 1.04–1.34; *p* = 0.010), MCS (OR 1.22; 95% CI 1.01–1.46; *p* = 0.032), AKI (OR 1.71; 95% CI 1.57–1.86; *p* < 0.001), and RRT (OR 2.22; 95% CI 1.87–2.63; *p* < 0.001). Hispanic patients had higher unadjusted odds of RRT (OR 1.79; 95% CI 1.46–2.19; *p* < 0.001), but lower odds of VT/VF (OR 0.65; 95% CI 0.55–0.76; *p* < 0.001) and MCS (OR 0.75; 95% CI 0.58–0.96; *p* = 0.023). Asian/Pacific Islander patients demonstrated the highest unadjusted odds across several severe in‐hospital complications, including cardiogenic shock (OR 2.14; 95% CI 1.71–2.68), MCS (OR 2.22; 95% CI 1.64–3.02), AKI (OR 1.66; 95% CI 1.40–1.97), RRT (OR 2.57; 95% CI 1.85–3.56), and stroke (OR 1.71; 95% CI 1.13–2.58), all *p* < 0.001. Patients categorized as Other race/ethnicity had higher unadjusted odds of cardiogenic shock (OR 1.30; 95% CI 1.04–1.62; *p* = 0.019) (Table 2). Resource utilization outcomes, including length of stay, total hospital charges, and disposition, are reported in Supporting Information S1: Table 2.

#### 3.2.2 Adjusted Analysis

**FIGURE 2:**
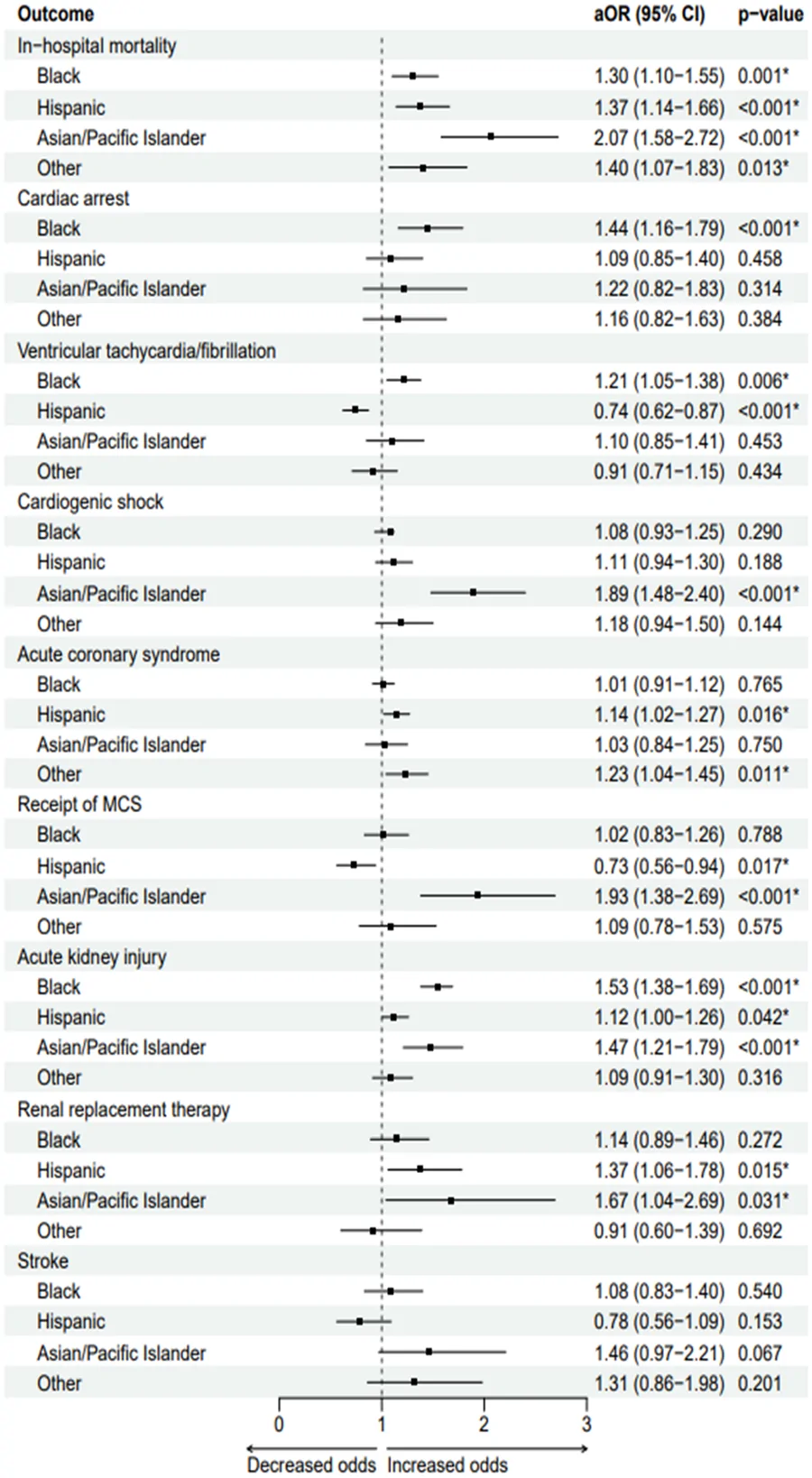
Adjusted odds ratio for outcomes compared to White patients. Forest plot showing adjusted odds ratios and 95% confidence intervals for in‐hospital mortality and secondary outcomes by race and ethnicity, with White patients as the reference group. The vertical reference line indicates an adjusted odds ratio of 1.0. ACS, acute coronary syndrome; AKI, acute kidney injury; aOR, adjusted odds ratio; CI, confidence interval; MCS, mechanical circulatory support; RRT, renal replacement therapy; VT/VF, ventricular tachycardia/fibrillation.

After multivariable adjustment, compared with White patients, in‐hospital mortality was significantly higher among Black patients (aOR 1.30; 95% CI 1.10–1.55; *p* < 0.001), Hispanic patients (aOR 1.37; 95% CI 1.14–1.66; *p* < 0.001), Asian/Pacific Islander patients (aOR 2.07; 95% CI 1.58–2.72; *p* < 0.001), and patients categorized as Other race/ethnicity (aOR 1.40; 95% CI 1.07–1.83; *p* = 0.013). The largest adjusted mortality disparity was observed among Asian/Pacific Islander patients (Figure 2).

Among secondary outcomes, Black patients had significantly higher adjusted odds of cardiac arrest (aOR 1.44; 95% CI 1.16–1.79; *p* < 0.001), VT/VF (aOR 1.21; 95% CI 1.05–1.38; *p* = 0.006), and AKI (aOR 1.53; 95% CI 1.38–1.69; *p* < 0.001). However, after adjustment, there were no significant differences in cardiogenic shock, ACS, MCS, RRT, or stroke among Black patients relative to White patients.

For Hispanic patients, adjusted analyses showed higher odds of ACS (aOR 1.14; 95% CI 1.02–1.27; *p* = 0.016), AKI (aOR 1.12; 95% CI 1.00–1.26; *p* = 0.042), and RRT (aOR 1.37; 95% CI 1.06–1.78; *p* = 0.015), but lower odds of VT/VF (aOR 0.74; 95% CI 0.62–0.87; *p* < 0.001) and MCS (aOR 0.73; 95% CI 0.56–0.94; *p* = 0.017). There were no significant differences in cardiac arrest, cardiogenic shock, or stroke after adjustment.

Asian/Pacific Islander patients had the most severe adjusted risk profile. In addition to the highest adjusted odds of mortality, they had significantly higher odds of cardiogenic shock (aOR 1.89; 95% CI 1.48–2.40; *p* < 0.001), MCS (aOR 1.93; 95% CI 1.38–2.69; *p* < 0.001), AKI (aOR 1.47; 95% CI 1.21–1.79; *p* < 0.001), and RRT (aOR 1.67; 95% CI 1.04–2.69; *p* = 0.031). Differences in cardiac arrest, VT/VF, ACS, and stroke were not statistically significant after adjustment.

Patients categorized as Other race/ethnicity had higher adjusted odds of ACS (aOR 1.23; 95% CI 1.04–1.45; *p* = 0.011), with no significant differences in other secondary outcomes (Figure 2). Complete‐case sensitivity analysis without multiple imputation showed similar findings and is reported in Supporting Information S1: Table 3.

#### 3.2.3 Mortality Trends

**FIGURE 3:**
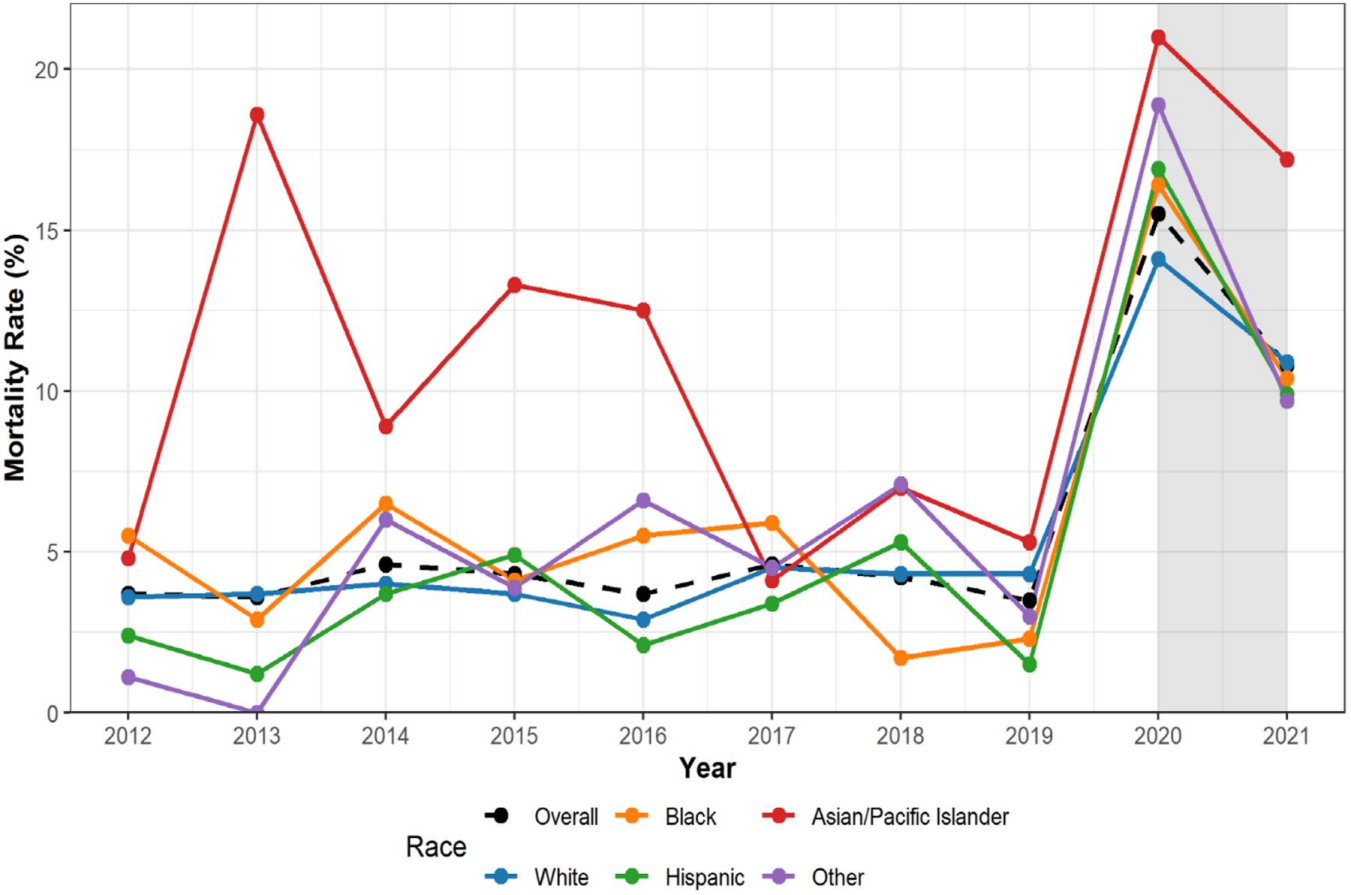
Trends in racial disparities for mortality (2012–2021). Annual weighted in‐hospital mortality rates among adults hospitalized with acute myocarditis in the United States from 2012 to 2021, stratified by race and ethnicity. The shaded region represents the COVID‐19 pandemic era. Abbreviation: COVID‐19, coronavirus disease 2019.

Figure 3 illustrates the temporal trends of in‐hospital mortality across all racial subgroups over the study period. During the pre‐pandemic era (2012–2019), the overall unadjusted mortality rate remained relatively stable, hovering between 3.5% and 4.6%.

Following the onset of the COVID‐19 pandemic, a sharp, universal increase in absolute mortality was observed across all races. This absolute spike was visually most pronounced among Asian/Pacific Islander patients, whose raw mortality rate peaked at 21% in 2020, as well as 18.9% among Other race patients. While raw mortality rates slightly decreased for all groups in 2021, they remained elevated above pre‐pandemic baselines.

#### 3.2.4 Pandemic Era Analysis

To determine whether the COVID‐19 pandemic modified racial and ethnic disparities in myocarditis mortality, we performed stratified pre‐pandemic and pandemic‐era analyses with interaction testing. The overall interaction between race/ethnicity and pandemic era was not statistically significant (p for interaction = 0.072), suggesting that the magnitude of racial and ethnic differences in in‐hospital mortality did not significantly change during 2020–2021 (Table 3).

**TABLE 3:** Adjusted odds ratios for in‐hospital mortality by race and ethnicity in pre‐COVID and post‐COVID eras.

Race	Pre‐COVID Era (2012–2019), aOR (95% CI)	Post‐COVID Era (2020–2021), aOR (95% CI)	*p* value for interaction
Black	1.26 (0.97–1.64)	1.26 (1.00–1.58)	0.990
Hispanic	0.99 (0.71–1.37)	1.41 (1.11–1.78)	0.080
Asian/Pacific Islander	2.57 (1.78–3.72)	1.57 (1.06–2.34)	0.044
Other	1.12 (0.73–1.70)	1.50 (1.04–2.17)	0.382

*Note:* Adjusted odds ratios are shown relative to White patients as the reference group. Values are presented as aORs with 95% CIs. Pre‐COVID era was defined as 2012–2019 and post‐COVID era as 2020–2021. *p* values for interaction evaluate whether the association between race/ethnicity and in‐hospital mortality differed by era.

Abbreviations: aOR, adjusted odds ratio; CI, confidence interval; COVID‐19, coronavirus disease 2019.

In stratified analyses, Black patients maintained higher odds of mortality compared with White patients across both the pre‐pandemic (aOR 1.26; 95% CI 0.97–1.64) and pandemic eras (aOR 1.26; 95% CI 1.00–1.58). Hispanic patients had similar mortality odds to White patients before the pandemic (aOR 0.99; 95% CI 0.71–1.37), but higher odds during the pandemic era (aOR 1.41; 95% CI 1.11–1.78); however, the interaction did not reach statistical significance (p for interaction = 0.080). Similarly, patients classified as Other race/ethnicity had a numeric increase in mortality odds during the pandemic era (aOR 1.50; 95% CI 1.04–2.17) compared with the pre‐pandemic era (aOR 1.12; 95% CI 0.73–1.70), although this change was not statistically significant (p for interaction = 0.382). In contrast, the mortality disparity among Asian/Pacific Islander patients significantly narrowed during the pandemic era compared with the pre‐pandemic period (p for interaction = 0.044) (Table 3).

#### 3.2.5 Sensitivity Analysis Excluding COVID‐19

To assess the impact of COVID‐19 diagnoses on these findings, we conducted a sensitivity analysis excluding all hospitalizations with a COVID‐19 diagnosis. After exclusion, the higher adjusted mortality observed among Hispanic patients in the primary analysis was attenuated (aOR 0.94; 95% CI 0.71–1.24; *p* = 0.682). In contrast, higher adjusted mortality persisted among Black patients (aOR 1.29; 95% CI 1.04–1.62; *p* = 0.020) and Asian/Pacific Islander patients (aOR 2.45; 95% CI 1.78–3.37; *p* < 0.001) (Supporting Information S1: Table 4). Excluding COVID‐19 hospitalizations also blunted the absolute mortality spike observed in 2020 across most racial groups (Supporting Information S1: Figure 1).

## 4 Discussion

In this nationally representative analysis of 105,235 adult hospitalizations for acute myocarditis in the United States, we identified significant racial and ethnic disparities in in‐hospital outcomes that persisted after adjustment for demographics, socioeconomic variables, and comorbidities. This study yields three principal findings. First, all racial and ethnic minority groups had significantly higher adjusted in‐hospital mortality compared with White patients, with Asian/Pacific Islander patients having the greatest risk. Second, the pattern of complications differed across groups, characterized primarily by an arrhythmic phenotype in Black patients and a hemodynamic phenotype in Asian/Pacific Islander patients. Third, Hispanic patients experienced higher mortality alongside lower observed use of MCS. These findings build on our group's prior 2011–2019 NIS analysis, while extending the study period through 2021 to include the COVID‐19 pandemic era and providing a more comprehensive assessment of racial and ethnic differences in mortality and major in‐hospital complications [15].

Differences in baseline characteristics across racial and ethnic groups likely contribute to the disparities in in‐hospital outcomes. Black and Hispanic patients were hospitalized with myocarditis at younger ages than White patients, a pattern consistent with findings from the Get With the Guidelines‐Heart Failure registry, which showed earlier age at first heart failure hospitalization among Black, Hispanic, and Asian patients [16] Black populations also carry disproportionately high rates of hypertension, diabetes mellitus, and CKD, driven in part by longstanding structural and social inequities [17]. In our cohort, Black and Hispanic patients were more likely to be insured through Medicaid and to reside in lower‐income ZIP code quartiles, socioeconomic markers that have been consistently associated with worse cardiovascular outcomes. Neighborhood disadvantage has been independently associated with higher in‐hospital mortality after MI [18], and the 2024 American College of Cardiology Expert Consensus on Myocarditis identified social determinants of health as an important but understudied contributor to outcome disparities in this condition [19].

Asian/Pacific Islander patients had the highest in‐hospital mortality in the cohort. This excess risk coincided with the highest adjusted odds of cardiogenic shock and MCS use. This occurred despite a socioeconomic profile not typically associated with neighborhood‐level disadvantage, as these patients were more likely to carry private insurance and reside in the highest income quartile. Prior work has also shown higher in‐hospital mortality among Asian patients in other acute cardiovascular settings, including acute myocardial infarction complicated by COVID‐19 and cardiogenic shock [20, 21].

Several mechanisms may contribute to this pattern. In a national analysis of cardiogenic shock hospitalizations from 2002 to 2019, Asian patients experienced higher in‐hospital mortality than non‐Asian patients, supporting the possibility that severe cardiovascular injury in this population may be more likely to progress along a hemodynamically unstable trajectory [21]. Prior studies have also described distinct cardiometabolic risk profiles among Asian populations, including greater visceral and hepatic adiposity relative to overall body mass and higher prevalence of elevated lipoprotein(a) [22, 23, 24]. Using UK Biobank data, Patel et al. demonstrated that South Asian populations had more than twice the adjusted hazard of atherosclerotic cardiovascular disease compared with Europeans, with residual risk persisting after accounting for a range of potential mediators [25]. Although acute myocarditis is distinct from atherosclerotic cardiovascular disease, these observations suggest that unmeasured cardiometabolic and inflammatory factors may contribute to more severe presentations in some Asian populations [7]; however, these mechanisms remain hypothesis‐generating.

Furthermore, the high prevalence of sepsis (24.5%) observed among Asian/Pacific Islander patients in our cohort may suggest a higher frequency of mixed septic and cardiogenic shock. This complex hemodynamic state, characterized by both distributive and pump‐failure components, can be particularly challenging to recognize and manage, potentially contributing to the more severe presentation and higher mortality observed in this population.

Black patients had a distinct complication pattern, with significantly higher adjusted odds of cardiac arrest, VT/VF, and AKI. The finding is consistent with broader epidemiologic literature demonstrating a disproportionate burden of SCD and malignant ventricular arrhythmias among Black individuals. In the Atherosclerosis Risk in Communities study, Black participants had a significantly higher lifetime risk of SCD than White participants, with events occurring at younger ages and influenced in part by socioeconomic factors such as income and education [26]. Similarly, in patients with nonischemic cardiomyopathy who had already undergone implantable cardioverter‐defibrillator (ICD) implantation, Younis et al. demonstrated that Black patients experienced a significantly higher adjusted risk of ventricular tachyarrhythmias and appropriate ICD therapies compared with White patients, a finding attributable to a higher arrhythmic burden rather than differential access to care [27]. In acute myocarditis, myocardial inflammation may interact with a preexisting arrhythmogenic substrate or greater adverse remodeling, increasing susceptibility to malignant electrical complications. Despite this higher arrhythmic burden, registry data show that Black patients remain less likely to be counseled, evaluated, or treated with advanced electrophysiologic therapies [28]. These disparities in care may further contribute to worse outcomes.

Hispanic patients showed a different pattern: despite significantly higher adjusted in‐hospital mortality, they had lower adjusted odds of VT/VF and less likely to receipt MCS, together with higher odds of AKI and RRT. Consistent with previously published data, Hispanic patients, particularly Hispanic women, bear a disproportionate mortality burden in STEMI complicated by cardiogenic shock (STEMI‐CS). In the largest NIS analysis (159 339 patients, 2006–2015), Hispanic women had the highest adjusted in‐hospital mortality of any race‐sex subgroup (aOR 1.46; 95% CI 1.26–1.70 vs. White men), followed by Black women (aOR 1.29) and White women (aOR 1.09) [29]. The International Society for Heart and Lung Transplantation/Heart Failure Society of America (ISHLT/HFSA) guideline on acute MCS similarly notes that among AMI‐CS patients in NIS data from 2003 to 2010, in‐hospital mortality was highest in patients with Hispanic ethnicity [30]. Within our cohort, higher mortality, together with lower observed use of MCS, raises concern for delayed recognition of hemodynamic compromise or differences in the escalation of care. The simultaneous excess of AKI and RRT is compatible with worse outcomes [31] and greater end‐organ hypoperfusion, although the present data cannot establish the mechanism directly.

Recent reviews of cardiogenic shock inequities describe lower use of MCS support among racial and ethnic minority populations [32]. In the REVIVAL study of ambulatory heart failure (HF) patients with access to ventricular assist device (VAD) centers, Black race was associated with lower VAD/transplant utilization, even after adjustment for heart failure severity, quality of life, social determinants of health, and patient preferences for care [33]. Limited English proficiency has also been associated with challenges in cardiovascular communication and care delivery, which may be especially relevant in rapidly evolving syndromes such as acute myocarditis [34]. Structural barriers and implicit bias may also influence timely recognition, referral, and escalation of care, including decisions related to cardiac catheterization, advanced therapies, and transplantation [35].

We also examined whether the COVID‐19 pandemic modified these disparities. The interaction between race and the pandemic era regarding overall mortality was not statistically significant, suggesting that the magnitude of racial and ethnic differences in myocarditis mortality did not change significantly during 2020 to 2021. The attenuation of the relative mortality gap among Asian/Pacific Islander patients should be interpreted cautiously and may reflect changes in case mix, admission thresholds, or relative increases in mortality across comparison groups during the pandemic. In a sensitivity analysis excluding COVID‐19 diagnoses, the excess mortality odds among Hispanic patients were attenuated, suggesting that COVID‐19 diagnoses may have contributed substantially to the observed mortality disparity in this group. This aligns with epidemiologic data showing that Hispanic communities experienced high rates of severe COVID‐19 infection and mortality, likely exacerbating baseline risks associated with acute myocardial inflammation [36, 37]. In contrast, higher adjusted mortality persisted among Black and Asian/Pacific Islander patients after excluding COVID‐19 cases, suggesting that these disparities were not solely explained by COVID‐19 hospitalizations.

This study should be interpreted in light of several limitations. As a retrospective analysis of an administrative database, it is subject to selection bias, coding error, and residual confounding. Case identification relied on ICD codes, which may have led to misclassification of myocarditis hospitalizations. Although ICD codes may capture broad myocarditis categories, the NIS lacks granular clinical data to adjudicate myocarditis etiology, severity, inflammatory profile, imaging findings, biopsy results, or Asian/Pacific Islander subgroup identity. In addition, certain demographic and socioeconomic variables are imperfectly captured in administrative datasets, and race and ethnicity were not self‐reported but recorded by hospital personnel, introducing potential misclassification. Because the NIS is a hospitalization‐level database, it does not permit evaluation of longitudinal outcomes or repeated admissions at the individual patient level. Nonetheless, the large nationally representative sample remains a key strength of this study and provides important insight into racial and ethnic disparities in myocarditis outcomes across the United States.

## 5 Conclusion

In this nationally representative analysis of patients hospitalized with myocarditis, significant racial and ethnic disparities in in‐hospital mortality and major complications persisted after multivariable adjustment. All racial and ethnic minority groups had higher adjusted odds of in‐hospital mortality compared with White patients, with Asian/Pacific Islander patients carrying the greatest excess risk. Asian/Pacific Islander patients demonstrated the highest in‐hospital mortality and a predominantly hemodynamic complication profile. Complication patterns differed meaningfully across groups, with Black patients disproportionately affected by arrhythmic events and Hispanic patients exhibiting excess mortality alongside lower observed use of mechanical circulatory support. These findings support further investigation into the structural, clinical, and biologic determinants of differential outcomes across racial and ethnic groups, as well as efforts to improve equity in the care of patients hospitalized with acute myocarditis.

## Author Contributions

MFO, PLO, and SVA contributed to the conception and design of the study, data analysis, interpretation of the findings, and preparation of the manuscript. MD, LV, and JC contributed to the interpretation of the findings and critically revised the manuscript for important intellectual content. RC supervised the study, contributed to its design and interpretation, and critically revised the manuscript. All authors reviewed and approved the final version of the manuscript and agree to be accountable for all aspects of the work.

## Funding

The authors have nothing to report.

## Ethics Statement

This study used the publicly available, de‐identified National Inpatient Sample and was therefore exempt from institutional review board review. The investigation was conducted in accordance with the ethical principles of the Declaration of Helsinki.

## Consent

Patient consent was not required because this study used a de‐identified administrative database. No copyrighted or previously published material requiring permission was reproduced in this manuscript.

## Conflicts of Interest

The authors declare no conflicts of interest.

## Supporting information


Supporting File

## Data Availability

The data that support the findings of this study are available on request from the corresponding author. The data are not publicly available due to privacy or ethical restrictions. The data that support the findings of this study are available from the Healthcare Cost and Utilization Project National Inpatient Sample (HCUP‐NIS), Agency for Healthcare Research and Quality, subject to the standard HCUP data use agreement.
