# Supplementary material for: Racial and Ethnic Disparities in Acute Myocarditis Outcomes in the United States
Source: Clin Cardiol. 2026 Aug 31;49(9):e70460. doi: 10.1002/clc.70460 (PMC13530382; doi:10.1002/clc.70460)
Supplement: Supplementary file 1 [file CLC-49-e70460-s001.docx]

**Supplementary Table 1. ICD-9-CM and ICD-10-CM/PCS Codes Used to Identify Diagnoses and Procedures**

| **Variables** | **ICD-9-CM Codes** | **ICD-10-CM/PCS Codes** |
| --- | --- | --- |
| Myocarditis | 42290, 07423, 42291, 42292, 42293, 42299, 4220, 4290 | B3322, I40.X, I41, I514, J1082, J1182 |
| Hypertension | 401.X, 402.X, 403.XX, 404.XX, 405.XX | I10, I11, I12, I13, I15, I158, I159, I1A0 |
| Obesity | 278.00, 278.01, 278.03 | E66.01, E66.09, E66.1, E66.2, E66.8, E66.9 |
| Diabetes Mellitus | 250.X | E08.X, E09.X, E10.X, E11.X, E12.X, E13.X, E14.X |
| COPD | 490, 491.X, 492.X, 496 | J41.X, J42, J43.X, J44.X, E88.01 |
| Coronary artery disease | 412.X, 414.0X, 414.2, 414.3, 414.4, 414.8, 414.9 | I25.1X, I25.2, I25.6, I25.8X, I25.9 |
| Chronic kidney disease | 585.1, 585.2, 585.3, 585.4, 585.5, 585.9 | N18.1, N18.2, N18.3X, N18.4, N18.5, N18.9 |
| End Stage Renal Disease | 585.6, V45.11 | N18.6, Z99.2 |
| Peripheral artery disease | 443.9, 440.X | I70.X, I73.9 |
| Chronic heart failure | 428.0, 428.1, 428.22, 428.32, 428.42, 428.23, 428.33, 428.43, 428.9, 40211, 40291, 40411, 40413, 40491, 40493 | I50.1, I50.2, I50.20, I50.22, I50.23, I50.3, I50.30, I50.32, I50.33, I50.4, I50.40, I50.42, I50.43, I50.81, I50.810, I50.812, I50.813, I50.814, I50.9, I110, I130, I132, I50.84 |
| Atrial fibrillation | 427.31 | I48.0, I48.1, I48.11, I48.19, I48.2, I48.20, I48.21, I48.91 |
| Chronic liver disease (Cirrhosis) | 571.2, 571.5, 571.6, 572.3, 572.2 | K70.3, K74.6, K74.3, K74.4, K74.5 |
| Malignancy | 140 - 199, 200 - 209, 230 - 234, 235 - 239 | C00 - C96, D00 - D09, D37 - D49 |
| Sepsis | 038.XX, 995.91, 995.92 | A40.XX, A41.XX, R65.20 |
| Anemia | 282.X, 283.0, 283.10, 283.19, 283.9, 284.01, 284.09, 284.81, 284.89, 284.9, 285.0, 285.21, 285.22, 285.29, 285.8, 285.9, | D46.4, D50.X, D51.X, D51.0, D52.X, D53.X, D55.X, D56.X, D57.X, D58.X, D59.0, D59.1, D59.10, D59.11, D59.12, D59.13, D59.19, D59.2, D59.4, D59.8, D59.9, D60.X, D61.01, D61.02, D61.09, D61.1, D61.2, D61.3, D61.89, D61.9, D62, D63.0, D63.1, D63.8, D64.X, O9903, O99011, O99012, O99013, O99019 |
| DVT/PE | 415.11, 415.13, 415.19, 451.81, 451.9, 451.11,  451.19, 451.2, 453.40, 453.41, 453.42, 453.8, 453.9 | I82.491, I82.492, I82.493, I82.499, I82.409, I82.621, I82.622, I82.629, I82.401, I82.402, I82.623, I82.419, I824Y1, I82.4Y2, I82.4Y3, I82.403, I82.4Z1, I82.4Z2, I82.4Z3, I82.429, I82.A19, I82.890, I82.423, I82.439, I82.449, I82.413, I82.411, I82.412, I82.A11, I82.A12, I82.C11, I82.C12, I82.433, I82.443, I82.A13, I82.432, I82.441, I82.442, I82.C19, I82.421, I82.422, I82.C13, I82.431, I82.B19, I82.B13, I82.B11, I82.B12, I82.611, I82.612, I82.220, I82619, I82.613, I82.90, I82.4Y9, I82.4Z9, I82.649, I82.3, I81, I82.210, T8172XA, I82601, I82.602, I82.603, I97.89, I82.609, I82.451, I82.451, I82.452, I82.453, I82.459, I82.461, I82.462, I82.290, I26.99, I26.93, I26.02, I26.09, I26, I260, I2609, I269 |
| Acute kidney injury | 584.XX, 586, 997.5 | N17.X, N19, N99.0 |
| Renal replacement therapy | 39.55, 54.98 | 5A1D00Z, 5A1D60Z. 5A1D70Z, 5A1D80Z, 5A1D90Z |
| Mechanical circulatory support | IABP: 37.61  ECMO: 39.65  BiVAD: 37.52  Short term pMCS: 37.60, 37.62, 37.65, 37.68  LVAD: 37.66 | IABP: 5A02110, 5A02210, 5A02115, 5A02215  ECMO: 5A15223, 5A1522F, 5A1522G, 5A15A2F, 5A15A2G, 5A15A2H  BiVAD: 02RK0JZ, 02RL0JZ  Short term pMCS: 02HA0RS, 02HA3RS, 02HA4RS, 02HA0RZ, 02HA3RZ, 02HA4RZ, 5A02116, 5A02216, 02HA0RJ, 02HA3RJ, 02HA4RJ, 5A0211D, 5A0221D  LVAD: 02HA0QZ, 02HA3QZ, 02HA4QZ |
| Cardiac arrest | 427.5, 798.1, 798.2 | I46.X, I97.12XX, I97.71X, O0336, O0386, O0486, O0737, O0881, O2911X |
| Ventricular tachycardia/fibrillation | 427.1, 427.41, 427.42 | I47.2X, I49.01, I49.02 |
| Cardiogenic shock | 785.51, 998.01 | R57.0, T81.11 |
| Acute coronary syndrome | 410.X, 411.1 | I20.0, I21.X, I22.X |
| Stroke | 430, 431.XX, 432.9, 433.XX, 434.XX, 435.X, 436 | I60.XX, I61.XX, I62.9, I63.X, |

Diagnosis and procedure codes were used to identify acute myocarditis, baseline comorbidities, in-hospital outcomes, and therapeutic interventions.
**Abbreviations:** BiVAD, biventricular assist device; COPD, chronic obstructive pulmonary disease; DVT/PE, deep vein thrombosis/pulmonary embolism; ECMO, extracorporeal membrane oxygenation; IABP, intra-aortic balloon pump; ICD-9-CM, International Classification of Diseases, Ninth Revision, Clinical Modification; ICD-10-CM, International Classification of Diseases, Tenth Revision, Clinical Modification; ICD-10-PCS, International Classification of Diseases, Tenth Revision, Procedure Coding System; LVAD, left ventricular assist device; pMCS, percutaneous mechanical circulatory support.

**Supplementary Table 2. Resource Utilization by Race and Ethnicity Among Adults Hospitalized With Acute Myocarditis**

| **Outcome** | **White** | **Black** | **Hispanic** | **Asian/Pacific Islander** | **Other** | **P value** |
| --- | --- | --- | --- | --- | --- | --- |
| **Length of stay** | 3 (2-6) | 4 (2-8) | 3 (2-7) | 4 (2-10) | 4 (2-8) | <0.001 |
| **Total hospital charges ($)** | 41,696 (23,991- 86,103) | 49,074  (26,866- 98,987) | 55,607  (31,320- 116,352) | 72,804 (33,431- 163,016) | 54,082 (28,613- 122,595) | <0.001 |
| **Disposition** | | | | | |  |
| Routine | 44,959 (69.8%) | 12,555 (68.2%) | 10,454 (73.9%) | 1,954 (58.5%) | 3,385 (68.4%) | <0.001 |
| Transfer to the short-term hospital | 3,975 (6.2%) | 1,074 (5.8%) | 784 (5.5%) | 279 (8.4%) | 354 (7.1%) |  |
| Transfer to Skilled Nursing Facility (SNF), Intermediate Care Facility (ICF), or Another Type of Facility | 5,000 (7.8%) | 1,329 (7.2%) | 800 (5.7%) | 295 (8.8%) | 365 (7.4%) |  |
| Home Health Care (HHC) | 5,569 (8.6%) | 1,730 (9.4%) | 945 (6.7%) | 375 (11.2%) | 400 (8.1%) |  |
| Against Medical Advice (AMA) | 715 (1.1%) | 360 (2.0%) | 170 (1.2%) | 25 (0.7%) | 90 (1.8%) |  |
| Died | 4,144 (6.4%) | 1,325 (7.2%) | 990 (7.0%) | 410 (12.3%) | 365 (7.4%) |  |

Values are presented as median (interquartile range) for continuous variables and n (%) for categorical variables. Missing values for resource utilization outcomes were excluded. The category “discharge alive, destination unknown” is not reported in accordance with HCUP reporting guidance because it included cells with fewer than 10 hospitalizations.
**Abbreviations:** AMA, against medical advice; HHC, home health care; HCUP, Healthcare Cost and Utilization Project; ICF, intermediate care facility; SNF, skilled nursing facility.

**Supplementary Table 3. Adjusted Odds Ratios for In-Hospital Outcomes by Race and Ethnicity Without Multiple Imputation**

| **Outcome** | **Black** | **P value** | **Hispanic** | **P value** | **Asian/Pacific Islander** | **P value** | **Other** | **P value** |
| --- | --- | --- | --- | --- | --- | --- | --- | --- |
| **Primary outcome** | | | | | | | | |
| In-hospital mortality | 1.28 (1.08-1.52) | 0.004 | 1.37 (1.14-1.65) | <0.001 | 2.07 (1.57-2.72) | <0.001 | 1.40 (1.06-1.84) | 0.015 |
| **Secondary outcomes** | | | | | | | | |
| Cardiac arrest | 1.45 (1.17-1.80) | <0.001 | 1.12 (0.87-1.43) | 0.370 | 1.19 (0.79-1.79) | 0.390 | 1.17 (0.83-1.65) | 0.365 |
| Ventricular tachycardia/fibrillation | 1.18 (1.03-1.35) | 0.018 | 0.73 (0.62-0.87) | <0.001 | 1.09 (0.85-1.41) | 0.466 | 0.92 (0.72-1.17) | 0.506 |
| Cardiogenic shock | 1.08 (0.93-1.25) | 0.299 | 1.12 (0.95-1.32) | 0.158 | 1.94 (1.52-2.47) | <0.001 | 1.19 (0.93-1.51) | 0.151 |
| Acute coronary syndrome | 1.00 (0.91-1.11) | 0.872 | 1.15 (1.03-1.29) | 0.011 | 1.04 (0.85-1.26) | 0.694 | 1.26 (1.07-1.48) | 0.006 |
| Receipt of MCS | 1.01 (0.82-1.25) | 0.881 | 0.73 (0.57-0.95) | 0.021 | 1.92 (1.37-2.68) | <0.001 | 1.14 (0.81-1.59) | 0.436 |
| Acute kidney injury | 1.52 (1.27-1.68) | <0.001 | 1.13 (1.01-1.27) | 0.027 | 1.46 (1.20-1.78) | <0.001 | 1.12 (0.93-1.34) | 0.204 |
| Renal replacement therapy | 1.18 (0.92-1.51) | 0.170 | 1.40 (1.07-1.81) | 0.012 | 1.58 (0.97-2.58) | 0.064 | 0.94 (0.61-1.46) | 0.806 |
| Stroke | 1.08 (0.83-1.40) | 0.565 | 0.79 (0.56-1.11) | 0.178 | 1.48 (0.98-2.24) | 0.060 | 1.25 (0.81-1.93) | 0.305 |

Adjusted odds ratios are shown relative to White patients as the reference group. Values are presented as aORs with 95% CIs. This complete-case sensitivity analysis excluded observations with missing values and did not use multiple imputation.
**Abbreviations:** aOR, adjusted odds ratio; CI, confidence interval; MCS, mechanical circulatory support.

**Supplementary Figure 1. Trends in racial disparities for mortality (2012-2021) excluding patients with COVID-19 diagnosis**

Annual weighted in-hospital mortality rates among adults hospitalized with acute myocarditis in the United States from 2012 to 2021 after excluding hospitalizations with a COVID-19 diagnosis. Lines represent yearly mortality rates stratified by race and ethnicity. The shaded region represents the COVID-19 pandemic era.
**Abbreviation:** COVID-19, coronavirus disease 2019.

**Supplementary Table 4. Adjusted Odds Ratios for In-Hospital Outcomes by Race and Ethnicity after excluding COVID-19 diagnosis**

| **Outcome** | **Black** | **P value** | **Hispanic** | **P value** | **Asian/Pacific Islander** | **P value** | **Other** | **P value** |
| --- | --- | --- | --- | --- | --- | --- | --- | --- |
| **Primary outcome** | | | | | | | | |
| In-hospital mortality | 1.29 (1.04-1.62) | 0.020 | 0.94 (0.71-1.24) | 0.682 | 2.45 (1.78-3.37) | <0.001 | 1.04 (0.72-1.49) | 0.824 |
| **Secondary outcomes** | | | | | | | | |
| Cardiac arrest | 1.36 (1.05-1.76) | 0.016 | 0.87 (0.63-1.22) | 0.443 | 1.31 (0.82-2.09) | 0.249 | 1.16 (0.79-1.73) | 0.432 |
| Ventricular tachycardia/fibrillation | 1.22 (1.05-1.41) | 0.007 | 0.72 (0.60-0.88) | 0.001 | 1.13 (0.86-1.49) | 0.360 | 0.90 (0.69-1.16) | 0.428 |
| Cardiogenic shock | 1.05 (0.90-1.23) | 0.497 | 0.99 (0.83-1.20) | 0.991 | 2.05 (1.58-2.65) | <0.001 | 1.23 (0.95-1.58) | 0.109 |
| Acute coronary syndrome | 1.01 (0.90-1.13) | 0.802 | 1.06 (0.94-1.20) | 0.309 | 1.03 (0.83-1.27) | 0.785 | 1.18 (0.99-1.41) | 0.054 |
| Receipt of MCS | 1.00 (0.80-1.24) | 0.999 | 0.70 (0.53-0.93) | 0.014 | 2.11 (1.49-2.99) | <0.001 | 1.10 (0.78-1.56) | 0.560 |
| Acute kidney injury | 1.38 (1.23-1.54) | <0.001 | 0.97 (0.85-1.11) | 0.742 | 1.42 (1.14-1.77) | 0.001 | 0.97 (0.80-1.18) | 0.790 |
| Renal replacement therapy | 0.97 (0.74-1.27) | 0.849 | 1.03 (0.79-1.33) | 0.811 | 1.25 (0.69-2.25) | 0.449 | 0.94 (0.66-1.35) | 0.776 |
| Stroke | 1.02 (0.76-1.36) | 0.886 | 0.74 (0.50-1.09) | 0.131 | 1.44 (0.89-2.31) | 0.133 | 1.52 (0.99-2.33) | 0.051 |

Adjusted odds ratios are shown relative to White patients as the reference group. Values are presented as aORs with 95% CIs. Hospitalizations with a COVID-19 diagnosis were excluded from this sensitivity analysis.
**Abbreviations:** aOR, adjusted odds ratio; CI, confidence interval; MCS, mechanical circulatory support.
